# Ensemble deep learning models for protein secondary structure prediction using bidirectional temporal convolution and bidirectional long short-term memory

**DOI:** 10.3389/fbioe.2023.1051268

**Published:** 2023-02-13

**Authors:** Lu Yuan, Yuming Ma, Yihui Liu

**Affiliations:** School of Computer Science and Technology, Qilu University of Technology (Shandong Academy of Sciences), Jinan, China

**Keywords:** protein secondary structure prediction, bidirectional temporal convolutional network, bidirectional long short-term memory, multi-scale BTCN, reverse prediction, fusing the features

## Abstract

Protein secondary structure prediction (PSSP) is a challenging task in computational biology. However, existing models with deep architectures are not sufficient and comprehensive for deep long-range feature extraction of long sequences. This paper proposes a novel deep learning model to improve Protein secondary structure prediction. In the model, our proposed bidirectional temporal convolutional network (BTCN) can extract the bidirectional deep local dependencies in protein sequences segmented by the sliding window technique, the bidirectional long short-term memory (BLSTM) network can extract the global interactions between residues, and our proposed multi-scale bidirectional temporal convolutional network (MSBTCN) can further capture the bidirectional multi-scale long-range features of residues while preserving the hidden layer information more comprehensively. In particular, we also propose that fusing the features of 3-state and 8-state Protein secondary structure prediction can further improve the prediction accuracy. Moreover, we also propose and compare multiple novel deep models by combining bidirectional long short-term memory with temporal convolutional network (TCN), reverse temporal convolutional network (RTCN), multi-scale temporal convolutional network (multi-scale bidirectional temporal convolutional network), bidirectional temporal convolutional network and multi-scale bidirectional temporal convolutional network, respectively. Furthermore, we demonstrate that the reverse prediction of secondary structure outperforms the forward prediction, suggesting that amino acids at later positions have a greater impact on secondary structure recognition. Experimental results on benchmark datasets including CASP10, CASP11, CASP12, CASP13, CASP14, and CB513 show that our methods achieve better prediction performance compared to five state-of-the-art methods.

## 1 Introduction

As a major research hotspot in bioinformatics, protein secondary structure prediction (PSSP) is undoubtedly an important task (Yang et al., 2018). The protein primary structure consists of a linear arrangement of amino acid residues (Kumar et al., 2020). The secondary structure is a specific spatial structure formed by the peptide chain curling or folding according to a certain rule. Further folding based on the secondary structure can form the tertiary structure. As a bridge connecting the primary and tertiary structures, the improvement of PSSP not only helps us understand the structure and function of proteins but also better predicts the tertiary structure (Wang et al., 2008; Yaseen and Li, 2014a; Wang et al., 2017). In addition, PSSP can also facilitate drug design. However, biological techniques for PSSP are time-consuming and expensive, so we can use computers and deep learning (LeCun et al., 2015) methods to improve secondary structure prediction.

Generally, the eight classes of protein secondary structure can be divided into G (helix), H (*α*-helix), I (*π*-helix), E (*β*-sheet), B (*β*-bridge), S (bend), T (turn) and C (coil) (Kabsch and Sander, 1983). Three classes of protein secondary structure can be formed by classifying H, G, and I as H (helix), E and B as E (strand), and other structures as C (coil) (Yaseen and Li, 2014b; Ma et al., 2018; Zhang et al., 2018). In recent years, the research on 3-state PSSP is more in-depth. However, it is important to obtain more abundant protein structural information about the 8-state secondary structure.

In the early days of research, machine learning methods such as support vector machines (Hua and Sun, 2001; Yang et al., 2011), neural networks (Qian and Sejnowski, 1988; Faraggi et al., 2012), and *k*-nearest neighbors (Salzberg and Cost, 1992; Bondugula et al., 2005) were widely used for PSSP. Furthermore, the PSIPRED server used two feedforward neural networks to predict secondary structure (McGuffin et al., 2000). The JPred4 server used the JNet algorithm to improve accuracy (Drozdetskiy et al., 2015). However, these methods cannot extract the global information in the sequence well.

With the development and improvement of deep learning in recent years, neural networks with deep architectures have achieved remarkable results in various fields. The deep learning method can not only reduce the computational complexity but also effectively utilize the extracted information to improve the prediction accuracy. The SSpro applied profiles, BRNN and structural similarity to PSSP (Magnan and Baldi, 2014). The SPIDER3 server used the LSTM-BRNN model for 3-state PSSP (Heffernan et al., 2017). The SPOT-1D used ResNet to improve the SPIDER3 server (Hanson et al., 2019). The SAINT combined the self-attention mechanism and the Deep 3I network to improve PSSP (Uddin et al., 2020). However, these methods have complex network structures and high computational costs. In addition, Zhou et al. proposed a supervised generative stochastic network to predict secondary structure (Zhou and Troyanskaya, 2014). The DeepCNF combined conditional neural fields and shallow neural networks for prediction (Wang et al., 2016). Wang et al. (2017) proposed a deep recurrent encoder-decoder network for classification. The Porter 5 classifier used multiple BRNNs for prediction (Torrisi et al., 2018). The DeepCNN used multi-scale convolution to extract secondary structure features (Busia and Jaitly, 2017). The NetSurfP-2.0 combined CNN and LSTM to extract local and long-range interactions (Klausen et al., 2019). These methods can improve PSSP performance, but they are not only insufficient for long-range feature extraction but also fail to establish a good balance between local features and long-range features.

In recent years, temporal convolutional network (TCN) (Bai et al., 2018) has achieved remarkable performance (Lea et al., 2017), while outperforming popular models such as recurrent neural networks in most fields. TCN can only extract unidirectional features, but secondary structure prediction is influenced by past and future amino acids. To this end, we propose a bidirectional TCN (BTCN) by improving TCN, which can extract bidirectional deep dependencies between amino acids. Due to the waste of hidden layer information in BTCN, we further propose a multi-scale BTCN (MSBTCN), which can not only extract bidirectional features but also better preserve the feature information of intermediate residual blocks. However, MSBTCN may also introduce unnecessary information.

For high-dimensional long protein sequences, most existing methods with deep architectures not only lack long-range feature extraction capability but also ignore deep dependencies. In addition, a single model cannot extract key information in complex residue sequences and has great limitations. Therefore, this paper proposes a novel deep learning model that uses BTCN, bidirectional long short-term memory (BLSTM) (Graves and Schmidhuber, 2005) network and MSBTCN to improve the accuracy of PSSP. In the proposed model, the BTCN module using the sliding window technique can extract bidirectional deep local dependencies in protein sequences. The BLSTM module can extract the global interactions between amino acid residues. The MSBTCN module can further capture bidirectional deep long-range dependencies between residues, while better fusing and optimizing features. Our method can effectively utilize longer-term bidirectional feature information to model complex sequence-structure relationships. Due to the close correlation between 3-state and 8-state PSSP, we also propose to fuse the features of 3-state and 8-state PSSP for classification based on the model. Furthermore, this paper compares our proposed six novel deep models for PSSP by combining BLSTM with TCN, reverse TCN (RTCN), multi-scale TCN (MSTCN), BTCN and MSBTCN, respectively. To evaluate the prediction performance of the model, we compare it with state-of-the-art methods on benchmark datasets. Experimental results show that our methods achieve better performance, which can effectively solve the shortcomings of incomplete and insufficient feature extraction.

The main contributions of this paper: 1) We propose BTCN by improving TCN, which can extract bidirectional deep dependencies in sequences. To enable BTCN to extract local features, we preprocess the sequences using a sliding window technique. 2) We further propose MSBTCN, which can not only extract bidirectional deep features between residues but also better preserve the information of hidden layers. 3) We propose a novel deep learning model using BTCN, BLSTM and MSBTCN, which outperforms five state-of-the-art methods and improves the prediction accuracy of secondary structure. 4) We propose multiple novel deep learning models by combining BLSTM with TCN, RTCN, MSTCN, BTCN, and MSBTCN respectively, which can effectively solve the disadvantage of low long-range dependency extraction ability in long sequences. 5) We experimentally demonstrate that the reverse prediction of secondary structure is superior to the forward prediction, suggesting that the recognition of secondary structure is more correlated with amino acids at later positions. 6) We experimentally demonstrate that the fusion of 3-state and 8-state PSSP features can further improve the prediction performance of the secondary structure, which also provides a new idea for PSSP.

## 2 Materials and methods

### 2.1 Bidirectional long short-term memory networks (BLSTM)

**FIGURE 1:**
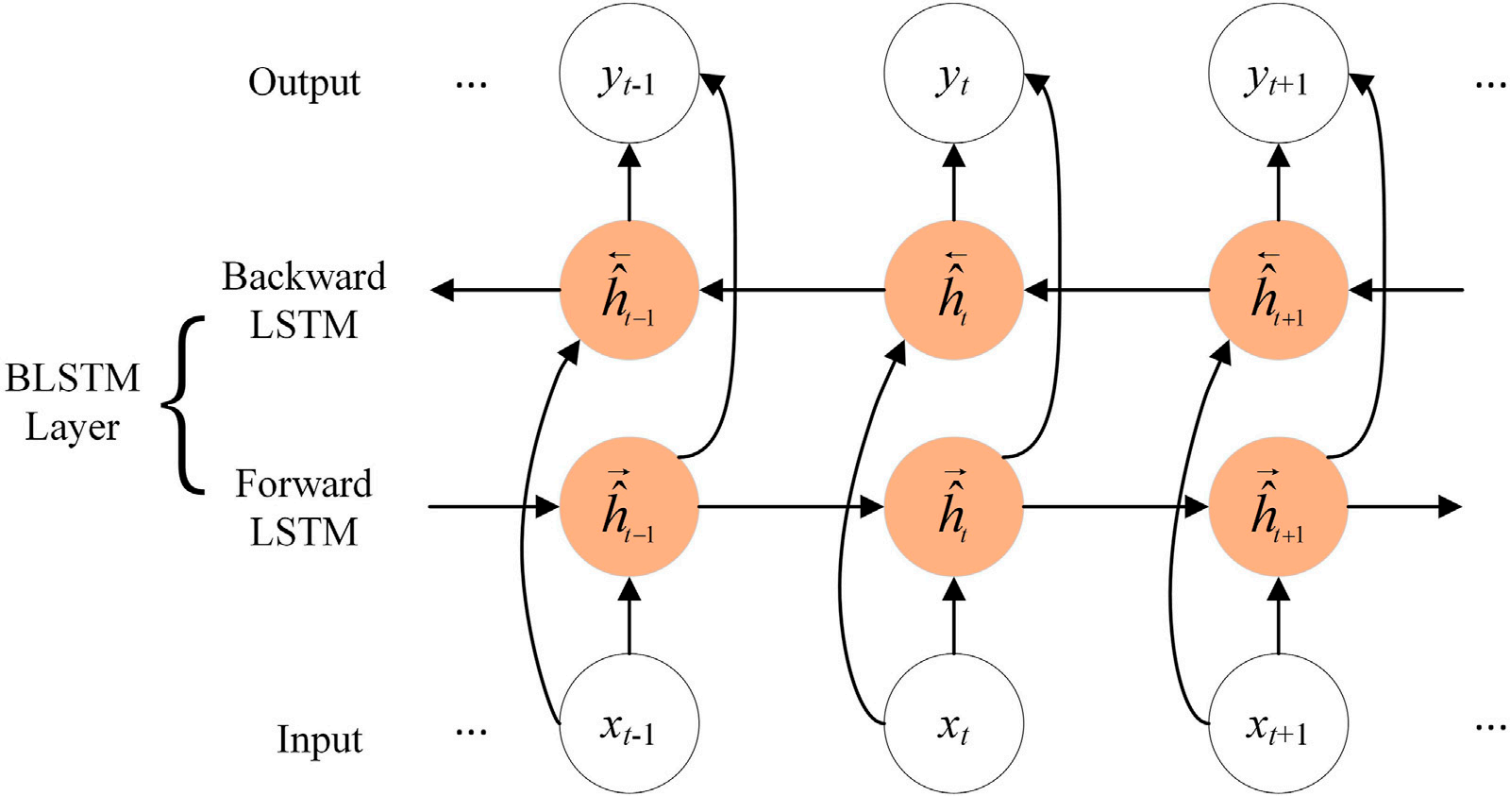
The architecture of BLSTM.

As shown in Figure 1, BLSTM consists of forward LSTM (Hochreiter and Schmidhuber, 1997) and backward LSTM. LSTM can automatically decide to discard unimportant information and retain useful information. For a standard LSTM cell at time *t*, the input feature is denoted as *x*_*t*_, the output is denoted as *h*_*t*_, and the cell state is denoted as *c*_*t*_. The forget gate *f*, the input gate *i* and the output gate *o* in the LSTM unit are calculated as follows:

$$f_{t}=\sigma \left(W_{xf}\times x_{t}+W_{hf}\times h_{t-1}+b_{f}\right)$$ (1)

$$i_{t}=\sigma \left(W_{xi}\times x_{t}+W_{hi}\times h_{t-1}+b_{i}\right)$$ (2)

$$o_{t}=\sigma \left(W_{xo}\times x_{t}+W_{ho}\times h_{t-1}+b_{o}\right)$$ (3)

$$c_{t}=f_{t}⊙c_{t-1}+i_{t}⊙\tanh\left(W_{xc}\times x_{t}+W_{hc}\times h_{t-1}+b_{c}\right)$$ (4)

$$h_{t}=o_{t}⊙\tanh\left(c_{t}\right)$$ (5)

where *σ* is the sigmoid function, *W* is the weight matrix, *b* is the bias term, ☉ is the element-wise multiplication, and tanh is the hyperbolic tangent function.

### 2.2 Temporal convolutional networks (TCN)

TCN has superior performance in sequence processing while avoiding the gradient problem during training. In addition, TCN also has the characteristics of fast calculation speed, low memory, parallel operation and flexible receptive field.

#### 2.2.1 Causal convolutions

TCN uses a one-dimensional fully convolutional network architecture, where the length of the input layer is the same as the length of each hidden layer, and zero padding is added to keep the front and back layers the same length. Therefore, TCN can map sequences of any length to output sequences of the same length. Furthermore, the network uses causal convolution, where the output at the current time is only determined by the feature inputs at the current time and past time. Therefore, information in TCN does not leak from the future to the past.

#### 2.2.2 Dilated convolutions

However, causal convolution has inevitable limitations when dealing with sequences that require long-term historical information. Therefore, the network uses dilated convolution to increase the receptive field and obtain very long effective historical information, which is defined as:

Fs=X∗fds=∑i=0k−1fi⋅Xs−d⋅i (6)

Where *F*(*s*) is the dilated convolution operation, *x* is the input feature, *d* is the dilation factor, *f* is the filter, *s* is the element of the sequence, *k* is the filter size, and *s* − *d • i* represents the past direction. As the number of layers and the dilation factor continue to increase (*d* = 2^*i*^ at level *i*), the output of the top layer will contain a wider range of input information.

#### 2.2.3 Residual connections

**FIGURE 2:**
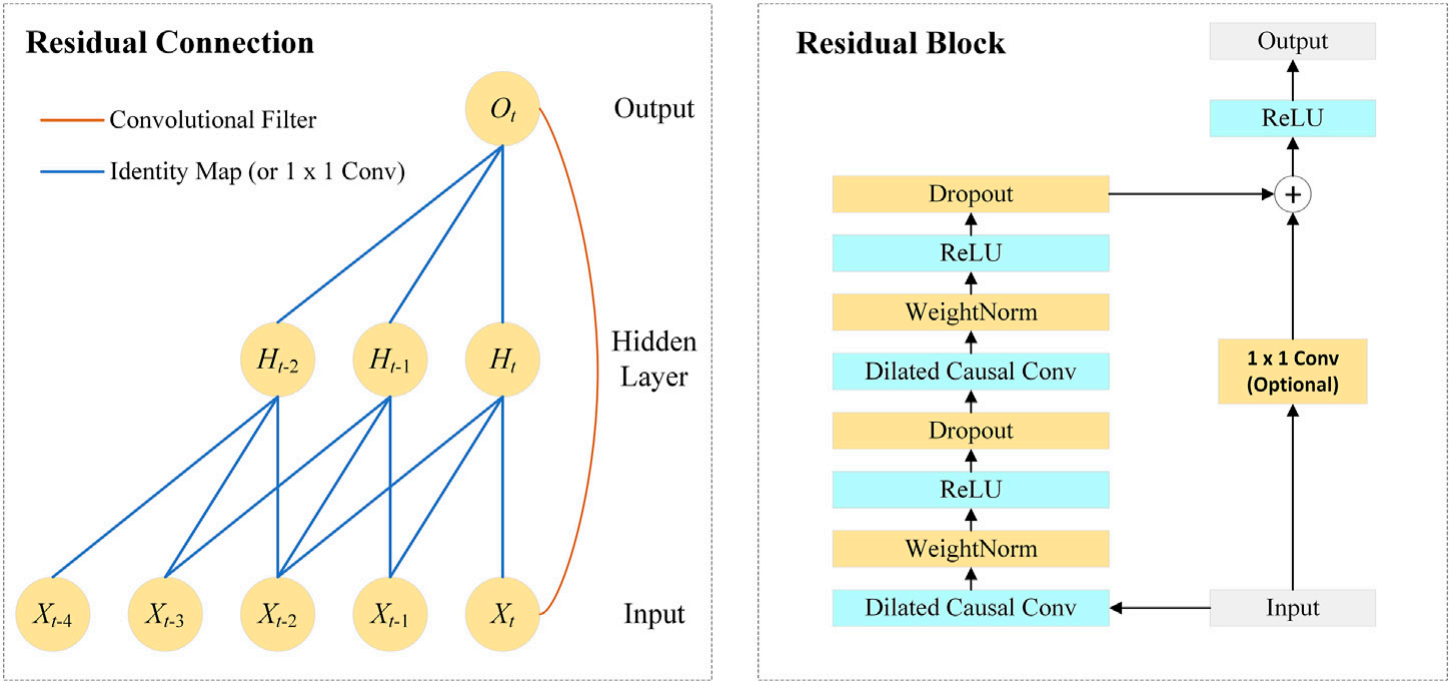
Architecture in TCN.

As shown in Figure 2, the network introduces residual connections to ensure the training stability of high-dimensional input, which is defined as:

$$O=Activation\left(X+F\left(X\right)\right)$$ (7)

where *X* represents the input of the block and *F*(*X*) represents the output of the block after a series of operations.

The TCN architecture consists of multiple residual blocks. As shown in Figure 2, the residual block contains dilated causal convolutional layers, weight normalization layers, ReLU layers, and dropout layers. The TCN adds the input of each block to the output of the block (including a 1 × 1 convolution on the input when the number of channels between the input and output do not match).

### 2.3 The proposed bidirectional TCN (BTCN)

Since TCN uses dilated causal convolution, it can only transmit information from the past to the future. However, the recognition of secondary structure is not only determined by amino acids at previous positions but also influenced by amino acids at later positions. The unidirectionally transported TCN obviously cannot satisfy the comprehensive extraction of amino acid features, so we propose a BTCN model to adequately capture the bidirectional deep dependencies between residues.

**FIGURE 3:**
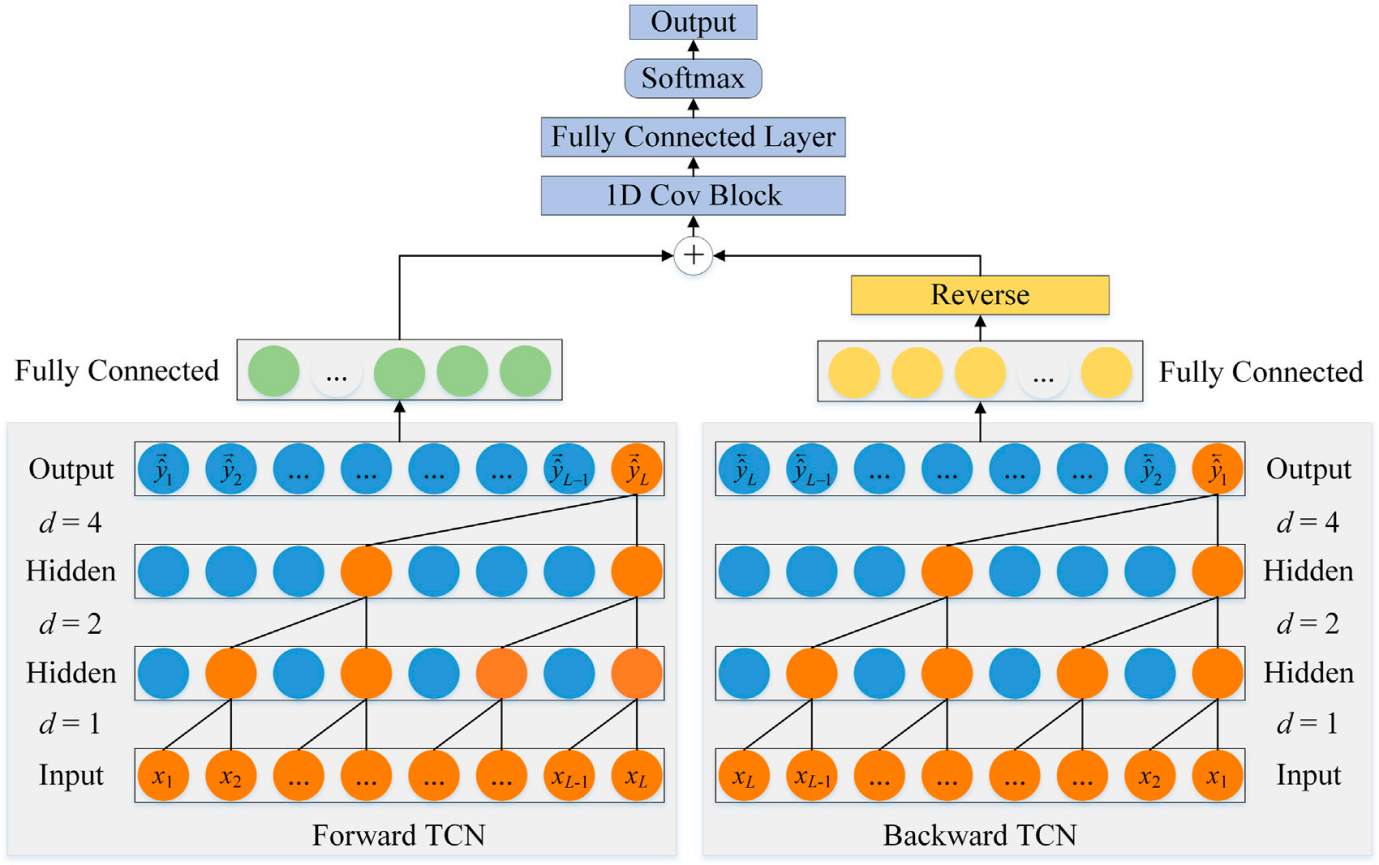
The architecture of BTCN.

As shown in Figure 3, the architecture of BTCN consists of forward TCN and backward TCN. Since the dilated causal convolution performs one-way operation on the sequence, we input the reverse sequence to the backward TCN for reverse feature extraction of the network.

Letting *X* denote a protein sequence, *L* be the length of *X*, and *X* = {*x*_1_, *x*_2_, ..., *x*_*L*_}, $\overset{⃖}{X}$ = {*x*_*L*_, *x*_*L*-1_, ..., *x*_1_} is reverse sequence of *X*. The BTCN can be expressed as follows:

$$\hat{Y}=\vec{TCN}\left(X\right)$$ (8)

$${\hat{Y}}_{1}=\overset{⃖}{TCN}\left(\overset{⃖}{X}\right)$$ (9)

$$Ouput=softmax\left(f\left(W\left(1DCov\left(\hat{Y}⊕\overset{⃖}{{\hat{Y}}_{1}}\right)\right)+b\right)\right)$$ (10)

where $\vec{TCN}$ is the forward TCN whose input is the forward sequence *X*, $\overset{⃖}{TCN}$ is the backward TCN whose input is the reverse sequence $\overset{⃖}{X}$ , ⊕ is the addition operation of the matrix, $\hat{Y}$ and ${\hat{Y}}_{1}$ are the outputs of the forward and backward TCN respectively, $\overset{⃖}{{\hat{Y}}_{1}}$ is the reverse matrix of ${\hat{Y}}_{1}$ , 1DCov is the 1D convolution operation of the residual block, *W* and *b* are the weight matrix and bias term of the fully connected layer, softmax is the activation function for classification, and *Output* is the final output of BTCN.

The output ${\hat{y}}_{t}$ of the network at the current time *t* can be determined by the input of the entire sequence, which is calculated as:

$${\hat{y}}_{t}=\vec{TCN}\left(x_{1},x_{2},\cdots ,x_{t}\right)⊕\overset{⃖}{TCN}\left(x_{L},x_{L-1},\cdots ,x_{t}\right)$$ (11)

$$Ouput_{t}=softmax\left(f\left(W\left(1DCov\left({\hat{y}}_{t}\right)\right)+b\right)\right)$$ (12)

We denote the forward $\vec{TCN}$ as TCN, because the input of the backward $\overset{⃖}{TCN}$ is the reverse sequence, so it is also called reverse TCN and denoted as RTCN. Therefore, the architecture of the network is BTCN = 1DCov(TCN + RTCN), where 1DCov can further optimize the features. In the network, TCN operates on inputs at times *t* and before *t* (*x*_1_, *x*_2_, … , *x*_*t*_), and RTCN operates on inputs at times *t* and after *t* (*x*_*L*_, *x*_*L*-1_, … , *x*_*t*_). Therefore, the network can utilize bidirectional deep interactions to facilitate secondary structure recognition through forward and backward extraction of residue features. Furthermore, BTCN is not limited to PSSP, it applies to all sequences that require global semantics.

### 2.4 The proposed multi-scale bidirectional TCN (MSBTCN)

In unidirectional TCN, the output ${\hat{y}}_{q}$ of the *q*th layer residual block is:

$${\hat{y}}_{q}=ReLU\left(f\left(W\times {\hat{y}}_{q-1}+b\right)+{\hat{y}}_{q-1}\right)$$ (13)

where the input ${\hat{y}}_{q-1}$ is the output of the previous layer. As the number of layers in BTCN increases, the receptive field of the network continues to expand. However, since BTCN adopts the dilated convolutional architecture, the hidden layer in the middle of the network loses a lot of important feature information. Therefore, we further propose the MSBTCN model to more comprehensively utilize residue features for classification. The improved MSBTCN can not only extract bidirectional multi-scale features but also better preserve the key information of the intermediate residual blocks.

**FIGURE 4:**
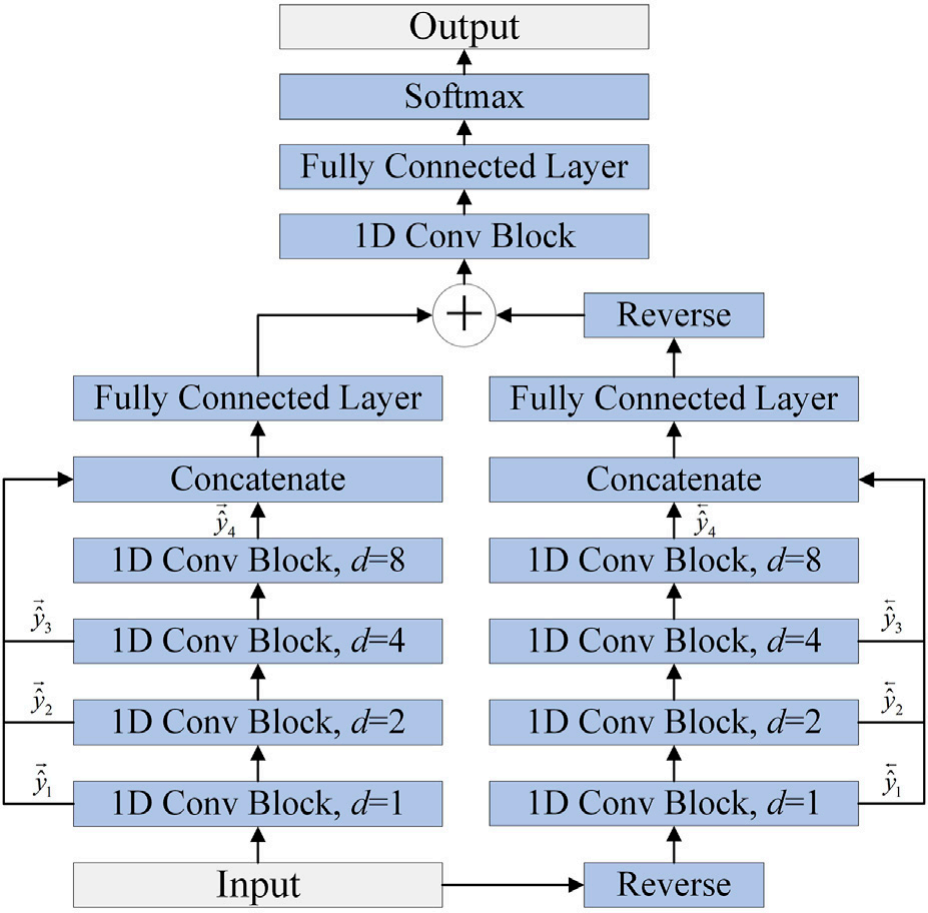
The architecture of MSBTCN.

As shown in Figure 4, the MSBTCN can utilize the complete information of all layers for prediction, which effectively prevents the waste of weight information in hidden layers. The output $\hat{y}$ of a unidirectional MSTCN with *n* residual blocks is:

$$\hat{y}=Concatenate\left({\hat{y}}_{1},{\hat{y}}_{2},\cdots ,{\hat{y}}_{n}\right)$$ (14)

The improved MSBTCN can not only capture the bidirectional deep features but also utilize the key information of the intermediate residual blocks for prediction.

### 2.5 Overall architecture of the proposed model

**FIGURE 5:**
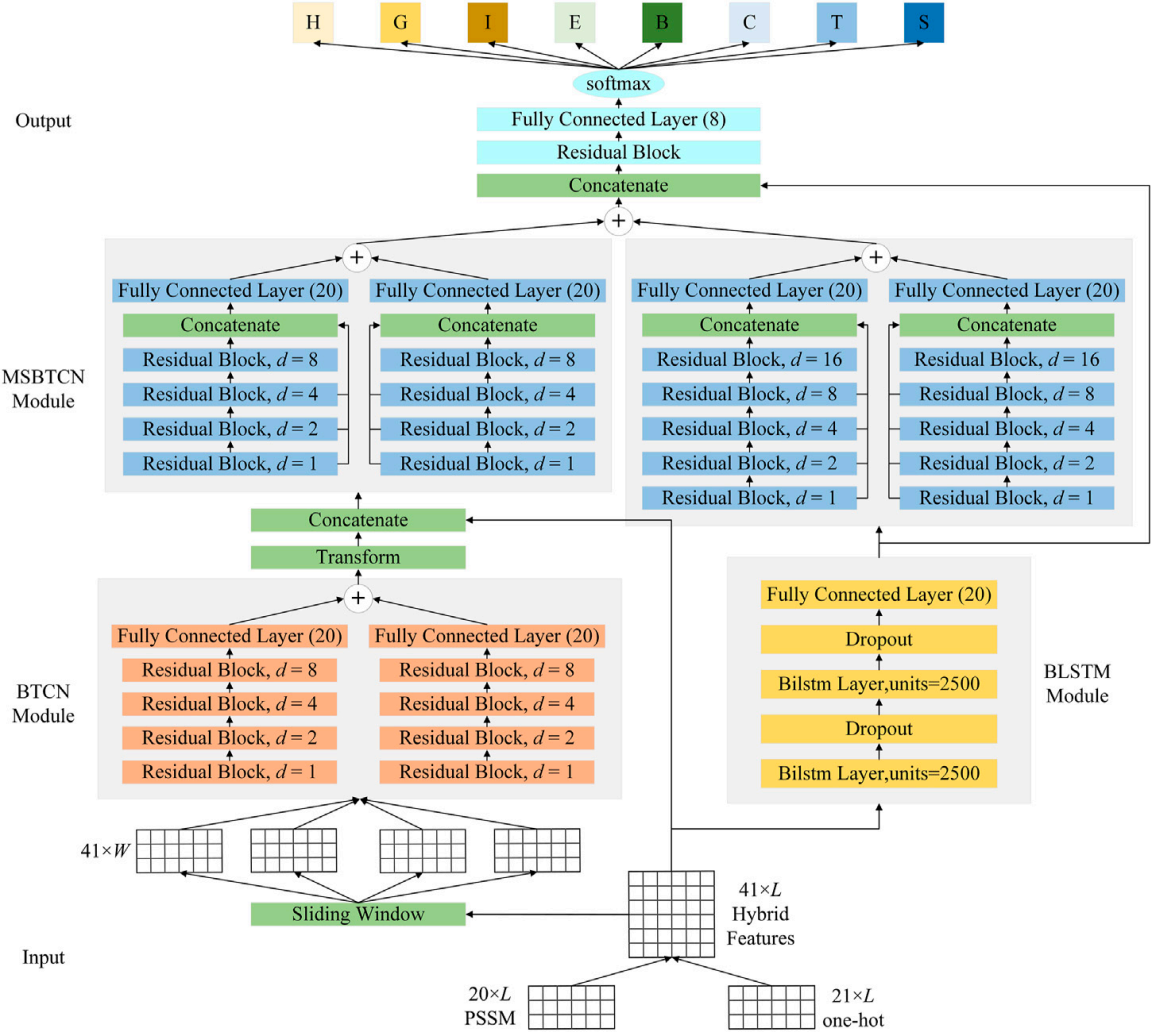
The detailed architecture of the proposed model.

To better improve the prediction of secondary structure, as shown in Figure 5, the proposed model uses BTCN, BLSTM and MSBTCN to extract deep interactions of residue sequences. The proposed model can be divided into five parts: input, BTCN module, BLSTM module, MSBTCN module and output.

In the input part, we first transform the protein data into 20-dimensional PSSM features and 21-dimensional one-hot features. Then, we use the hybrid feature PSSM + one-hot of size 41 × *L* as the input of the model, where *L* is the length of the protein sequence.

In the BTCN module, to enable the network to extract local features, we use the sliding window technique to segment the input features into short sequences of 41 × *W*, where *W* is the window size. The input and output of BTCN for sequence-to-sequence prediction must be the same length, so we put the secondary structure label corresponding to the segmented amino acid feature at the *W* position, and fill the remaining positions with 0. We then use the modified BTCN to extract bidirectional deep local dependencies in amino acid sequences. Since *W* is generally less than 20, our use of four residual blocks is sufficient to capture bidirectional amino acid information in the sequence. We use three dilated causal convolutional layers with the same dilation factor in the residual block. After the dilated causal convolutional layer, we add an instance normalization layer to accelerate model convergence, a ReLU activation layer to prevent vanishing gradient, and a spatial dropout layer to avoid model overfitting. We use the Transform layer to process the extracted local features into 20 × *L* sequences. Then, the Concatenate layer can merge the local features with the input features into 61 × *L* sequences.

In the BLSTM module, we use two bidirectional LSTM layers with powerful analytical capabilities to extract key global interactions in protein sequences. Additionally, we add two dropout layers to ensure gradient stabilization during training.

In the MSBTCN module, we use four and five residual blocks to optimize the extracted local and global features, respectively, while further capturing the deeper bidirectional long-range dependencies between amino acid residues, which can more comprehensively preserve the important information of the hidden layer. Since MSBTCN has a flexible receptive field and stable computation, it can interact and control sequence information more accurately, while quickly optimizing and fusing the extracted features.

In the output part, we use a residual block to process and optimize the features extracted by MSBTCN and BLSTM modules. Finally, we use a fully connected layer and softmax function to complete the classification.

It should be noted that we also extract the Concatenate layer features of the output part of the model in 3-state and 8-state PSSP respectively and fuse them into 80-dimensional features for secondary structure prediction, where the fused features are denoted as FF_3-8_. The FF_3-8_ contains both 3-state and 8-state secondary structure label information, which can better model the sequence-structure mapping relationship between input features and secondary structures. The proposed method can effectively exploit more complex and longer-term global dependencies to improve the accuracy of PSSP through comprehensive processing of protein sequences.

## 3 Experiments

### 3.1 Datasets

The PISCES (Wang and Dunbrack, 2005) server can produce lists of sequences from the Protein Data Bank (PDB) based on chain-specific criteria and mutual sequence identity, which are widely used for PSSP. Therefore, we selected 14,991 proteins from the PDB to compose the CullPDB (Wang and Dunbrack, 2005) dataset based on the percentage identity cutoff of 25%, the R-factor cutoff of 0.25, and the resolution cutoff of 3 Å. To ensure the accuracy of the 8-state secondary structure information, we use the division method of the DSSP (Kabsch and Sander, 1983) program. We removed proteins that were duplicated with the test set in the training set. In addition, we also removed proteins with lengths less than 40 or more than 800. The final CullPDB dataset contains 14,562 protein chains. For better evaluation, we further randomly divide the dataset into three parts: a training set (11,650), a validation set (1,456) and a test set (1,456). The results of all experiments are obtained from the average of three times independent experiments.

To evaluate the performance of the proposed model, we also use the CASP10 (Moult et al., 2014), CASP11 (Moult et al., 2016), CASP12 (Moult et al., 2018), CASP13 (Kryshtafovych et al., 2019), CASP14 (Kryshtafovych et al., 2021), and CB513 (Cuff and Barton, 1999) datasets as test sets, where the numbers of proteins and residues in the six benchmark datasets are shown in Table 1. The first five datasets are from the Critical Assessment of Protein Structure Prediction (CASP) website https://predictioncenter.org/.

**TABLE 1:** The number of proteins and residues for the six datasets.

Number	CASP10	CASP11	CASP12	CASP13	CASP14	CB513
Proteins	99	81	19	22	23	513
Residues	24,048	20,084	4,257	5,948	4,644	84,119

### 3.2 Feature representation

In this study, we used two amino acid encoding methods: one-hot encoding and position-specific scoring matrix (PSSM) (Jones, 1999). The database of protein sequences contains 20 standard amino acid types (A, R, N, D, C, Q, E, G, H, I, L, K, M, F, P, S, T, W, Y, and V) and 6 non-standard amino acid types such as B, Z, and X. Since the occurrence frequency of the six non-standard amino acid types is particularly low, they can generally be classified as one type. Therefore, we consider that the protein sequence consists of 21 amino acid types.

An amino acid sequence of length *L* can be represented as a 21 × *L* feature matrix by one-hot encoding, where 21 represents the number of amino acid types, the position corresponding to the amino acid type is 1, and the other positions are 0. Each amino acid type in one-hot encoding has an independent number, which makes the vector representations of different amino acid types mutually orthogonal, so this method can also be called orthogonal encoding.

PSSM is a scoring matrix based on the alignment of the sequence itself with multiple sequences. This encoding method contains rich biological evolution information, so it is widely used for protein sequence representation in PSSP. In the experiments, PSSM was generated by PSI-BLAST (Altschul et al., 1997) with parameters including a threshold of 0.001 and 3 iterations. A 20 × *L* PSSM matrix represents a protein sequence of length *L*, where 20 is the number of standard amino acid types, that is, each row corresponds to one amino acid residue type.

### 3.3 Evaluation metrics

In this paper, we use four metrics to evaluate the performance of the proposed model: Q3 accuracy, Q8 accuracy and Segment overlap (Sov) (Zemla et al., 1999) score for 3-state and 8-state PSSP.

The 8-state secondary structure is H, G, I, E, B, S, T and C, while the 3-state secondary structure is H, E, and C. Q3 and Q8 accuracy are the ratios of the number of correct residues predicted to the number of all residues *S*, which are defined as:

$$Q3=\frac{S_{C}+S_{E}+S_{H}}{S}\times 100$$ (15)

$$Q8=\frac{S_{H}+S_{G}+S_{I}+S_{E}+S_{B}+S_{C}+S_{T}+S_{S}}{S}\times 100$$ (16)

where *S*_*i*_ (*i* ∈{*H*, *E*, *C*} or {*H*, *G*, *I*, *E*, *B*, *C*, *T*, *S*}) represents the correct number of predicted a single type *i*. Letting *S*_*S*_ denote the total number of residues of a single type *i*. The prediction accuracy *Q*_*i*_ of a single type *i* is defined as:

$$Q_{i}=\frac{S_{i}}{S_{S}}\times 100$$ (17)

Sov is a metric based on the ratio of overlapping segments, which is defined as:

$$Sov=\frac{100}{N_{Sov}}\underset{S_{0}}{\sum }\left[\frac{minov\left(S_{1},S_{2}\right)+\sigma \left(S_{1},S_{2}\right)}{maxov\left(S_{1},S_{2}\right)}length\left(S_{1}\right)\right]$$ (18)

where *N*_*Sov*_ is the total number of residues in the protein sequence, *S*_1_ is all observed structural segments, *S*_2_ is all predicted segments, *S*_0_ is all segments of *S*_1_ and *S*_2_ with the same structure, *length*(*S*_1_) is the residue length of *S*_1_, *maxov*(*S*_1_ *, S*_2_) is the union length of *S*_1_ and *S*_2_ segments, and *minov*(*S*_1_ *, S*_2_) is the intersection length of *S*_1_ and *S*_2_ segments. The factor *σ*(*S*_1_ *, S*_2_) allows variation at the segment edges, which is defined as:

$$\sigma \left(S_{1},S_{2}\right)=\min\left\{\begin{array}{c} maxov\left(S_{1},S_{2}\right)-minov\left(S_{1},S_{2}\right)\\ minov\left(S_{1},S_{2}\right)\\ \mathrm{int}\left[len\left(S_{1}\right)\right]/2\\ \mathrm{int}\left[len\left(S_{2}\right)\right]/2 \end{array}\right\}$$ (19)

### 3.4 Performance analysis of the proposed model

To make the proposed model have good performance when dealing with long protein sequences, we conduct extensive experiments on the CullPDB dataset without using FF_3-8_. For the three modules in the proposed model, we show and analyze the effect of different hyperparameters on the prediction performance in experiments.

#### 3.4.1 Effect of BTCN module parameters

**FIGURE 6:**
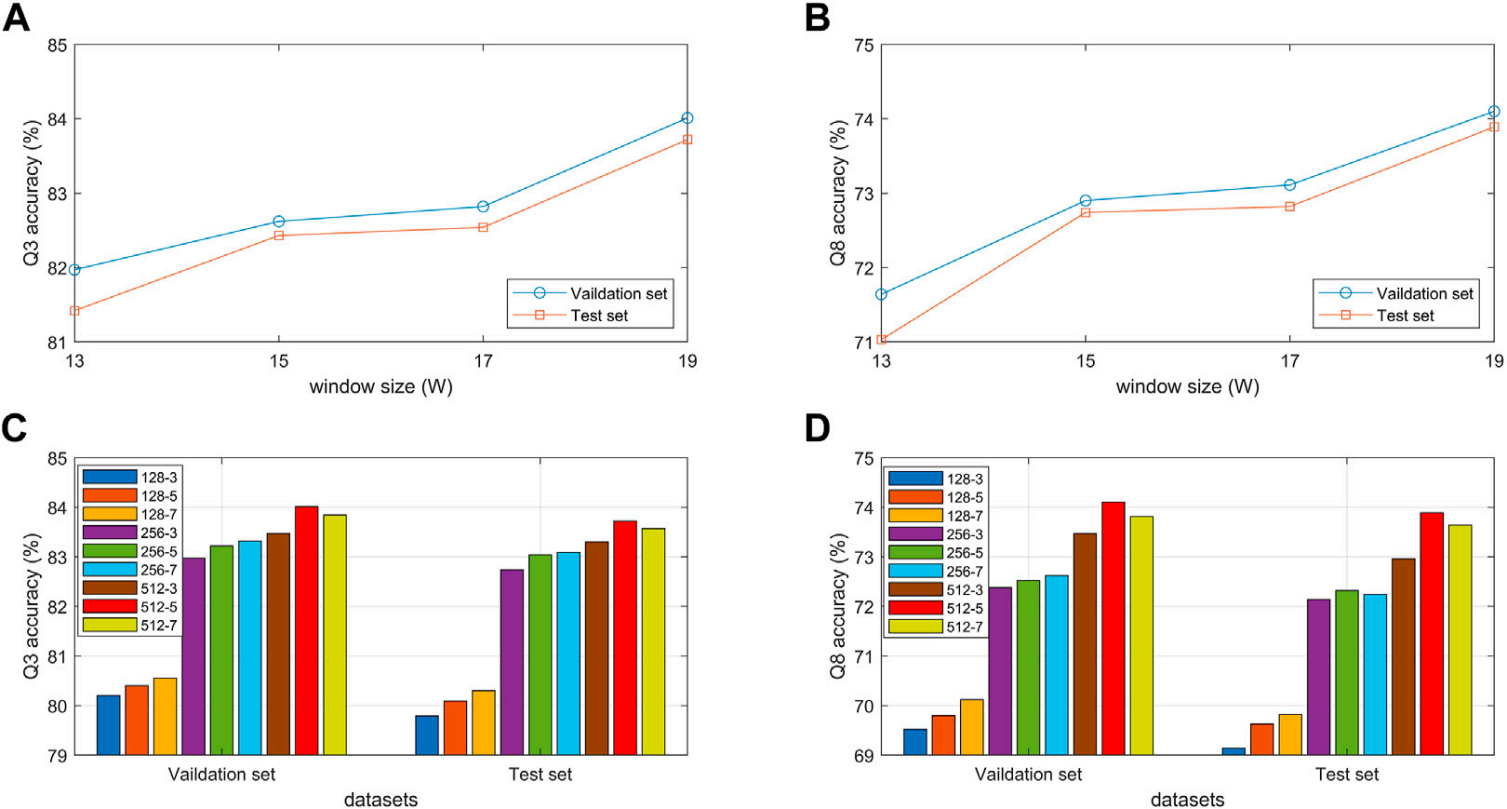
**(A,B)** Q3 and Q8 accuracy of the proposed model under different sliding window sizes. **(C,D)** Q3 and Q8 accuracy of the proposed model under different filter parameters.

To explore the effect of sliding window size and filter parameters on the proposed model, we conduct comparative experiments on validation and test sets. Since the recognition of secondary structure is mainly influenced by amino acids at current and adjacent positions, we used different sliding window sizes 13, 15, 17 and 19 to segment protein sequences. As shown in Figures 6A, B, when the sliding window size is 19, the model achieves the highest Q3 and Q8 accuracy on the two datasets. Because when the window is too small or too large, important amino acid information at key positions will be lost or ignored.

Figures 6C, D show the Q3 and Q8 accuracy of the proposed model under different numbers and sizes of filters. The figures show that when the number and size of filters are 512 and 5, the model achieves the best experimental results on the validation and test sets. The main reason is that the filter size determines the local extent of capture, which affects the extraction of key features between residues. Furthermore, the number of channels in the convolution not only affects the prediction performance but also determines the model size and training time.

#### 3.4.2 Effect of BLSTM module parameters

**FIGURE 7:**
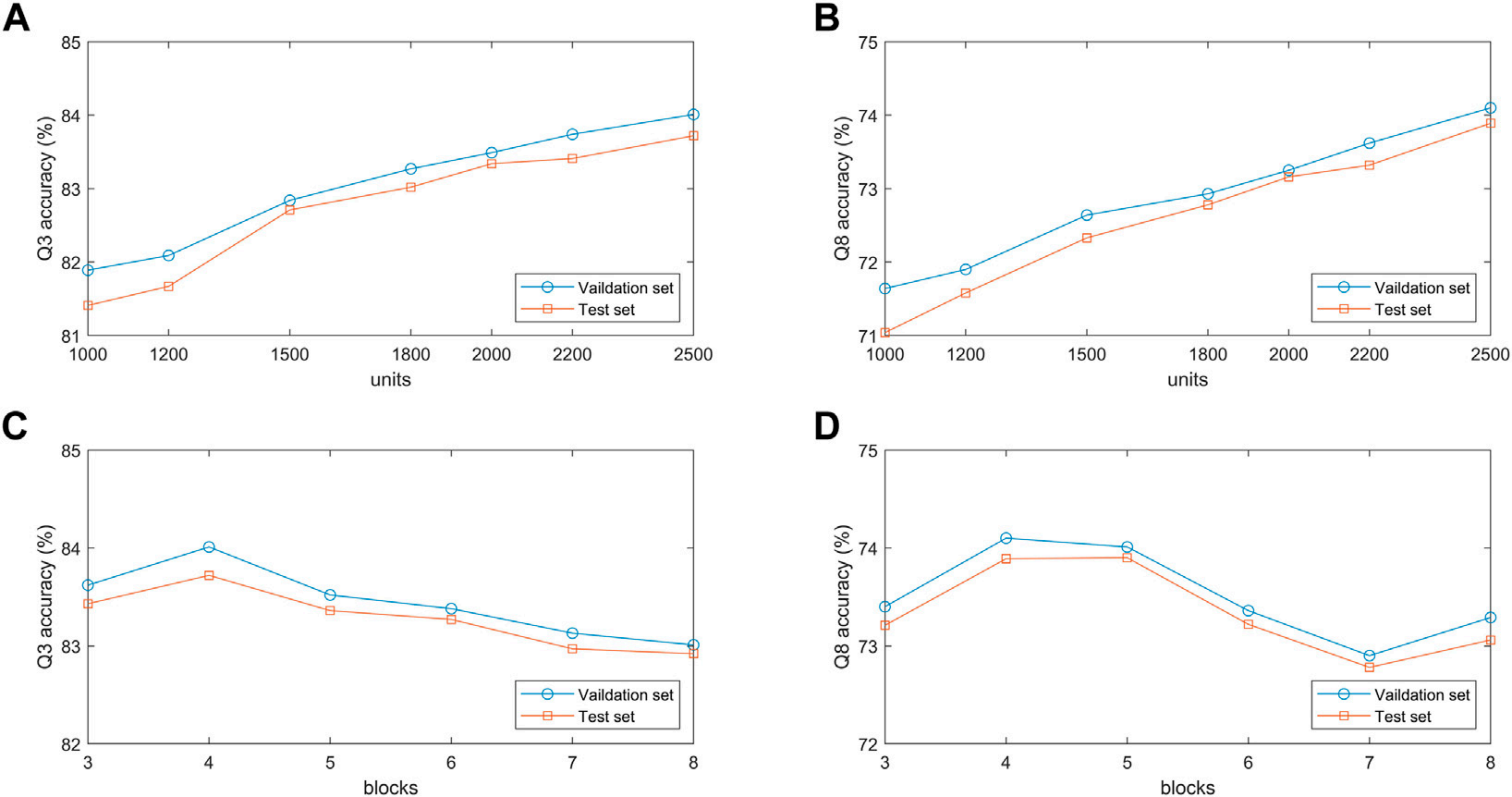
**(A,B)** Q3 and Q8 accuracy of the proposed model under different number of units. **(C,D)** Q3 and Q8 accuracy of the proposed model under different number of blocks.

To verify the effect of hidden units in the BLSTM layer on the proposed model, we conduct comparative experiments on the validation and test sets with different hidden unit numbers of 1,000, 1,200, 1,500, 1800, 2000, 2,200, and 2,500. Figures 7A, B show that the classification accuracy of the model on the two datasets increases as the number of hidden units increases. When the number of hidden units is 2,500, the model achieves the best prediction performance in 3-state and 8-state PSSP. The main reason is that the number of hidden units determines the expressivity of high-dimensional protein sequences. However, it should be noted that if the number of hidden units is too large, the model will not only slow down the training speed but also may have overfitting problems.

#### 3.4.3 Effect of MSBTCN module parameters

The performance of the MSBTCN module composed of residual blocks is closely related to the number of blocks, so we optimize the extracted local features with 3–8 different numbers of residual blocks, respectively. Figures 7C, D show the 3-state and 8-state accuracy of the proposed model on the validation and test sets. It can be observed that the Q3 accuracy on the two datasets reaches the maximum when the model uses 4 residual blocks, while the Q8 accuracy on the two datasets reaches the maximum when 4 and 5 blocks are used, respectively. Because the number of residual blocks determines the depth of our model. The model cannot capture deeper dependencies when the depth is not enough, but the model increases complexity and the risk of overfitting as the depth increases.

### 3.5 Ablation study

#### 3.5.1 Comparison of unidirectional and bidirectional PSSP

To demonstrate the effectiveness of the BTCN model for PSSP, we compare the performance of TCN, RTCN and BTCN on seven datasets. In the experiments, we use four residual blocks to extract features, where all models have the same parameters. The model input is the hybrid feature of size 41 × *L*, where *L* is the protein length. As shown in Table 2, the prediction performance of our BTCN is greatly improved on all datasets. Compared with TCN, the Q3 accuracy and Q8 accuracy of BTCN are improved by an average of 4.74% and 5.43% on seven datasets, respectively. The experimental results are sufficient to demonstrate the superior performance of the BTCN model, which can effectively capture the bidirectional deep interactions between residues and improve the prediction accuracy.

**TABLE 2:** Comparison of Q3 and Q8 accuracy of three models on seven datasets. Bold indicates the best performance.

Models	CullPDB		CASP10		CASP11		CASP12		CASP13		CASP14		CB513	
	Q3	Q8	Q3	Q8	Q3	Q8	Q3	Q8	Q3	Q8	Q3	Q8	Q3	Q8
TCN	75.61	63.91	77.78	65.50	75.72	63.43	76.13	63.94	75.95	61.95	74.68	62.77	80.09	66.38
RTCN	77.05	64.73	79.13	66.85	76.88	65.01	77.07	64.53	77.96	62.98	76.23	65.22	80.82	67.41
BTCN	**80.79**	**69.40**	**82.25**	**70.52**	**80.56**	**68.93**	**80.26**	**69.16**	**80.75**	**66.86**	**80.04**	**68.58**	**84.48**	**72.43**

**FIGURE 8:**
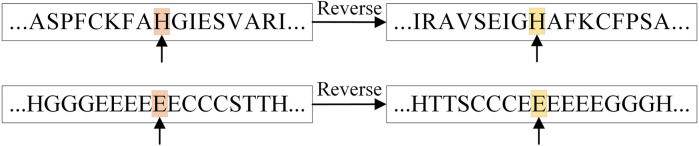
Reverse representation of amino acid and secondary structure sequences.

In addition, the table shows that the prediction accuracy of RTCN is consistently better than TCN on seven datasets and the Q8 accuracy is improved by an average of 1.18%. The reverse amino acid sequence is shown in Figure 8. The results show that the reverse prediction of the secondary structure is superior to the forward prediction, which also indicates that the amino acid at the later position has a greater impact on the overall recognition of the secondary structure when the features are extracted unidirectionally. The main reason for the low prediction accuracy of TCN is that its broad receptive field ignores the important information of adjacent amino acids when the sliding window technique is not used, but the prediction of the whole sequence can better reflect the influence of the amino acids in the front and rear positions on PSSP. The single-type prediction accuracy of TCN and RTCN is shown in Table 3. The table shows that the accuracy of types H, G, B, C, S and T has improved while the accuracy of type E has decreased on most datasets. This also demonstrates that the recognition of most secondary structure types is more relevant to amino acid information from later positions.

**TABLE 3:** Single-type accuracy comparison of TCN and RTCN on seven datasets.

Accuracy	CullPDB		CASP10		CASP11		CASP12		CASP13		CASP14		CB513	
	TCN	RTCN	TCN	RTCN	TCN	RTCN	TCN	RTCN	TCN	RTCN	TCN	RTCN	TCN	RTCN
QH	89.21	86.29	90.23	90.17	89.96	90.19	90.89	87.98	91.17	91.91	87.48	90.47	86.87	87.31
QG	12.45	16.94	18.15	22.48	7.79	8.72	8.15	14.81	6.84	10.27	10.81	7.43	30.80	33.34
QI	0	0	0	0	0	0	0	0	0	0	0	0	0	0
QE	80.41	76.84	73.92	78.90	82.86	78.00	80.15	76.99	77.64	72.27	72.08	75.45	77.40	77.26
QB	2.62	3.98	3.23	3.63	2.10	0	2.33	0	1.33	0	0	2.08	12.71	19.15
QC	53.14	58.25	65.61	58.28	50.76	58.86	51.56	61.30	58.40	60.34	57.17	56.84	61.23	62.32
QS	13.07	20.40	13.72	21.67	10.93	13.86	14.25	7.25	9.92	9.92	7.75	8.53	31.76	32.58
QT	36.22	43.34	45.95	51.10	35.99	44.18	36.34	43.98	27.72	39.84	30.97	38.28	48.52	52.12

#### 3.5.2 Comparison of the six proposed models

To verify the performance of different feature extraction modules in PSSP, we propose six novel deep learning models by combining BLSTM with TCN, RTCN, MSTCN, BTCN and MSBTCN, respectively. We use the same feature extraction process and parameters in all models. As shown in Table 4, the BLSTM-BTCN-MSBTCN model achieves the highest Q3 and Q8 accuracy on the eight datasets except CASP13. In addition, it can be observed that BLSTM-RTCN has better prediction performance on most datasets than BLSTM-TCN, which indicates that the reverse prediction of secondary structure can achieve higher accuracy. After the sequence is processed by the sliding window method, the effect of the amino acids in the front and rear positions on the prediction performance is not much different, so the advantage of RTCN is not obvious. The table shows that the prediction performance of the BLSTM-BTCN and BLSTM-MSBTCN models is significantly better than the models with unidirectional feature extraction on all datasets, which proves that our proposed BTCN and MSBTCN can fully exploit the bidirectional long-range interaction to improve the prediction accuracy. Although BLSTM-MSBTCN can capture multi-scale feature information, its prediction accuracy is inferior to BLSTM-BTCN on most datasets. The main reason is that BTCN does not waste the information of intermediate layers excessively when extracting local features in short sequences segmented by sliding windows, on the contrary, MSBTCN may introduce unnecessary information. To this end, we use BTCN and MSBTCN to extract bidirectional local and long-range features respectively in the BLSTM-BTCN-MSBTCN model, which can not only better extract features in short sequences but also preserve useful information in long sequences. The BLSTM-BTCN-MSBTCN model can combine the advantages of each module to extract diverse features and improve prediction accuracy.

**TABLE 4:** Comparison of 3-state and 8-state PSSP performance of the proposed six models on eight datasets. Validation and Test represent the validation set and test set of CullPDB, respectively. The six models have the same feature extraction process and parameters. Bold indicates the best performance.

Models	Validation		Test		CASP10		CASP11		CASP12		CASP13		CASP14		CB513	
	Q3	Q8	Q3	Q8	Q3	Q8	Q3	Q8	Q3	Q8	Q3	Q8	Q3	Q8	Q3	Q8
BLSTM-TCN	82.95	73.53	82.71	73.22	84.04	73.82	81.68	71.72	80.14	69.82	81.09	68.43	80.74	69.94	85.61	77.21
BLSTM-RTCN	83.03	73.60	82.87	73.34	83.96	73.74	81.53	71.59	80.32	70.08	81.17	68.50	80.71	70.10	85.68	77.27
BLSTM-MSTCN	83.05	73.64	82.83	73.29	84.01	73.86	81.79	71.75	80.39	69.73	80.94	68.29	80.63	70.05	85.73	77.18
BLSTM-BTCN	83.98	74.08	83.65	73.84	84.71	74.45	82.53	72.45	81.49	70.69	82.21	**69.04**	81.99	70.99	86.41	77.56
BLSTM-MSBTCN	83.93	74.03	83.68	73.79	84.77	74.48	82.47	72.51	81.43	70.51	82.16	68.96	81.83	70.87	86.45	77.71
BLSTM-BTCN-MSBTCN	**84.01**	**74.10**	**83.72**	**73.89**	**84.83**	**74.53**	**82.62**	**72.56**	**81.51**	**70.92**	**82.26**	69.02	**82.02**	**71.12**	**86.47**	**77.91**

### 3.6 Comparison with state-of-the-art methods

In this section, we compare the proposed model with five state-of-the-art models on seven datasets CullPDB, CASP10, CASP11, CASP12, CASP13, CASP14, and CB513 using Q3 accuracy, Q8 accuracy and Sov score as evaluation measures. Among the compared models, DCRNN (Li and Yu, 2016) is an end-to-end deep network that uses convolutional neural networks with different kernel sizes and recurrent neural networks with gated units to extract multi-scale local features and long-range dependencies in protein sequences. CNN_BIGRU (Drori et al., 2018) combines convolutional network and bidirectional GRU to predict secondary structure. DeepACLSTM (Guo et al., 2019) combines asymmetric convolutional networks and bidirectional long short-term memory networks to improve secondary structure prediction accuracy. These three algorithms are all combinations of convolutional neural networks and recurrent neural networks, but their structures are different. MUFOLD-SS (Fang et al., 2018) uses a Deep inception-inside-inception (Deep3I) network to handle local and global dependencies in sequences. ShuffleNet_SS (Yang et al., 2022) uses a lightweight convolutional network and label distribution aware margin loss to improve the network’s learning ability for rare classes. For a fair comparison, we use our dataset for training in experiments, where the input is the hybrid feature PSSM + one-hot.

The prediction results of the proposed methods and five existing popular methods DCRNN, CNN_BIGRU, DeepACLSTM, MUFOLD-SS, and ShuffleNet_SS on benchmark datasets are shown in Tables 5, 6. The table shows that our model consistently outperforms five state-of-the-art methods on seven datasets in terms of Q3 accuracy, Q8 accuracy and Sov scores for 3-state and 8-state PSSP. This is mainly attributed to the powerful and comprehensive feature extraction capability of the proposed model, which enables bidirectional deep local and long-range interactions in residue sequences to be fully extracted and used for prediction. Compared to our model without FF_3-8_, FF_3-8_ achieves the best 3-state PSSP performance on all datasets while FF_3-8_ also achieves the highest 8-state PSSP accuracy in most cases. The experimental results show that the important correlation between the 3-state and 8-state PSSP can mutually promote the recognition of secondary structure. In particular, the accuracy of the 3-state PSSP is significantly improved after adding the 8-state PSSP feature. Furthermore, our model size is 13.8 MB while the model size using FF_3-8_ is 14.3 MB. The model sizes of DCRNN, CNN_BIGRU, DeepACLSTM, MUFOLD-SS and ShuffleNet_SS are 18.1MB, 15.8MB, 20.6MB, 17.6 MB and 3.9MB, respectively. Although our model parameter size only outperforms four popular methods, it achieves state-of-the-art performance in PSSP. For high-dimensional long sequences, our model can also effectively utilize a broad and flexible receptive field to capture longer-term key dependencies between residues, so it can better model the complex relationship between sequence and structure.

**TABLE 5:** 3-state PSSP performance comparison with state-of-the-art methods on seven datasets. Bold indicates the best performance.

Methods	CullPDB		CASP10		CASP11		CASP12		CASP13		CASP14		CB513	
	Q3	Sov	Q3	Sov	Q3	Sov	Q3	Sov	Q3	Sov	Q3	Sov	Q3	Sov
DCRNN	82.12	78.51	82.57	75.71	80.57	75.53	80.41	74.75	80.49	77.09	80.28	71.46	84.66	79.63
CNN_BIGRU	82.31	78.68	82.40	76.20	81.03	76.58	80.37	75.62	80.64	76.94	80.45	71.92	84.81	79.90
DeepACLSTM	82.64	79.45	83.43	77.76	81.32	76.04	80.49	75.56	80.91	77.43	80.79	71.73	85.02	80.12
MUFOLD-SS	83.02	79.62	83.28	78.04	81.68	77.41	80.94	77.47	81.15	78.02	81.12	70.97	85.30	80.23
ShuffleNet_SS	83.07	78.79	83.89	76.27	81.72	76.37	80.87	76.39	81.43	77.46	81.26	71.32	85.62	79.98
Our Model	83.72	79.94	84.83	79.43	82.62	78.31	81.51	77.90	82.26	79.12	82.02	72.65	86.47	81.05
Our Model (FF_3-8_)	**84.45**	**81.24**	**85.83**	**80.97**	**83.51**	**80.12**	**82.01**	**79.18**	**82.72**	**79.66**	**83.44**	**75.51**	**87.49**	**83.12**

**TABLE 6:** 8-state PSSP performance comparison with state-of-the-art methods on seven datasets. Bold indicates the best performance.

Methods	CullPDB		CASP10		CASP11		CASP12		CASP13		CASP14		CB513	
	Q8	Sov	Q8	Sov	Q8	Sov	Q8	Sov	Q8	Sov	Q8	Sov	Q8	Sov
DCRNN	72.06	70.42	72.11	69.74	70.50	68.44	69.41	67.24	68.05	68.01	68.87	63.27	75.63	73.06
CNN_BIGRU	72.28	70.15	71.87	69.17	70.94	69.05	69.67	68.06	67.83	67.92	68.69	62.95	75.54	72.84
DeepACLSTM	72.86	71.34	73.09	71.42	71.24	69.93	69.82	68.18	68.47	69.31	69.52	63.46	76.01	73.46
MUFOLD-SS	73.32	71.59	72.98	71.51	71.62	69.84	70.23	69.32	68.23	68.89	69.23	62.69	76.64	74.04
ShuffleNet_SS	73.31	71.12	73.62	70.23	71.54	69.17	70.21	68.73	68.54	68.36	69.91	63.17	76.89	73.32
Our Model	73.89	72.50	74.53	72.25	72.56	70.90	**70.92**	**70.02**	69.02	69.78	71.12	64.32	77.91	74.96
Our Model (FF_3-8_)	**74.13**	**72.71**	**74.88**	**72.31**	**72.64**	**70.95**	**70.92**	68.70	**69.10**	**70.70**	**71.21**	**65.25**	**78.12**	**75.66**

### 3.7 The single-type accuracy of the 8-state PSSP

In 8-state PSSP, the single-type accuracy of the proposed model without and with FF_3-8_ on seven datasets is shown in Table 7. It can be seen from the table that there are obvious differences in the prediction accuracy of the eight structures. The main reason is that the frequency of occurrence of various types is too different, and the number of structure type I is almost 0. It can be observed that the accuracy of structure types G, E, B and T is improved on most datasets after adding the features of 3-state PSSP.

**TABLE 7:** Single-type accuracy of the proposed model on seven datasets in 8-state PSSP when using FF_8_ and FF_3-8_, where F_8_ represents the 8-state PSSP feature.

Accuracy	CullPDB		CASP10		CASP11		CASP12		CASP13		CASP14		CB513	
	FF_8_	FF_3-8_	FF_8_	FF_3-8_	FF_8_	FF_3-8_	FF_8_	FF_3-8_	FF_8_	FF_3-8_	FF_8_	FF_3-8_	FF_8_	FF_3-8_
QH	90.44	91.74	91.54	92.90	91.73	91.79	92.88	92.42	91.72	91.72	89.72	88.91	93.76	93.58
QG	35.47	38.97	34.66	35.71	35.80	29.85	36.30	39.26	27.00	28.14	30.41	33.11	52.27	54.05
QI	9.62	3.85	0	0	0	0	0	0	0	0	0	0	0	0
QE	83.29	83.46	85.50	85.64	83.43	82.72	80.52	81.54	80.76	78.77	79.14	79.87	86.88	87.65
QB	19.20	19.83	20.97	18.95	15.13	13.87	11.63	13.95	4.00	8.00	12.50	16.67	36.01	33.10
QC	69.29	68.39	71.80	74.40	65.45	69.91	62.34	61.07	65.46	68.15	65.23	65.01	74.19	72.06
QS	35.59	38.28	32.45	31.20	35.94	30.82	27.75	26.75	23.09	27.15	30.49	28.17	49.51	52.24
QT	61.19	57.46	63.62	59.32	55.73	56.14	54.40	55.56	51.81	46.30	49.46	52.90	63.70	65.86

## 4 Conclusion

In this paper, we propose a novel deep learning model for PSSP using BTCN, BLSTM and MSBTCN. In the proposed model, we use a modified BTCN module to extract bidirectional deep local dependencies in protein sequences segmented by the sliding window technique. Then, we use the BLSTM module to extract the global interactions between amino acids. We also use a modified MSBTCN module to further capture the bidirectional key long-range dependencies between residues while better optimizing and fusing the extracted features, which prevents information waste in hidden layers. The proposed model has strong stability and feature extraction ability, and it can not only effectively solve the shortcomings of insufficient extraction of deep long-range dependencies in sequences but also overcome the weaknesses of each module. Due to the close correlation between the 3-state and the 8-state, we also use the fusion feature FF_3-8_ based on the proposed model to further improve the performance of PSSP, which is also a new idea for PSSP. Moreover, this paper compares the six PSSP models we propose by combining BLSTM with TCN, RTCN, MSTCN, BTCN, and MSBTCN, respectively. In addition, we experimentally demonstrated that the reverse prediction of secondary structure can achieve higher accuracy, which indicates that amino acids at later positions are more correlated with secondary structure recognition than amino acids at earlier positions. We evaluate the performance of the proposed model on benchmark datasets such as CASP10, CASP11, CASP12, CASP13, CASP14, and CB513 using Q3 accuracy, Q8 accuracy and Sov score. Experimental results show that our method has better prediction performance compared to state-of-the-art methods. Our methods can fully use the diverse deep features in residue sequences for prediction to better model the complex mapping relationship between sequences and structures, thereby improving the accuracy of PSSP. Our models are not limited to PSSP but are applicable to all data that rely on bidirectional information. When dealing with other real sequence data, BTCN may ignore some information while MSBTCN may introduce unimportant information. Therefore, in the future, we will investigate more feature extraction and optimization techniques to better utilize protein information, and study the association between 3-state and 8-state PSSP to improve prediction accuracy.

## Data Availability

The original contributions presented in the study are included in the article/supplementary material, further inquiries can be directed to the corresponding authors.
